# Coenzyme Q10 and Its Therapeutic Potencies Against COVID-19 and Other Similar Infections: A Molecular Review

**DOI:** 10.34172/apb.2023.026

**Published:** 2021-11-07

**Authors:** Mohammad Fakhrolmobasheri, Mahnaz-Sadat Hosseini, Seyedeh-Ghazal Shahrokh, Zahra Mohammadi, Mohammad-Javad Kahlani, Seyed-Erfan Majidi, Mehrdad Zeinalian

**Affiliations:** ^1^Department of Genetics and Molecular Biology, School of Medicine, Isfahan University of Medical sciences, Isfahan, Iran.; ^2^School of Pharmacy and Pharmaceutical Sciences, Isfahan University of Medical Sciences, Isfahan, Iran.; ^3^Department of Biology, Faculty of Sciences, Shahid Bahonar University of Kerman, Kerman, Iran.; ^4^Department of Cell and Molecular Biology and Microbiology, Faculty of Biological Sciences and Technologies, University of Isfahan, Isfahan, Iran.; ^5^Pediatric Inherited Disease Research Center, Research Institute for Primordial Prevention of Non-Communicable Disease, Isfahan University of Medical Sciences, Isfahan, Iran.; ^6^Iranians Cancer Control Charity Institute (MACSA), Isfahan, Iran.

**Keywords:** Coenzyme Q10, Therapeutic, COVID-19, Molecular, Infection, Coronavirus

## Abstract

***Purpose:*** New lethal coronavirus disease 2019 (COVID-19), currently, has been converted to a disastrous pandemic worldwide. As there has been found no definitive treatment for the infection in this review we focused on molecular aspects of coenzyme Q_10_ (CoQ_10_) and possible therapeutic potencies of CoQ_10_ against COVID-19 and similar infections.

***Methods:*** This is a narrative review in which we used some authentic resources including PubMed, ISI, Scopus, Science Direct, Cochrane, and some preprint databases, the molecular aspects of CoQ_10_ effects, regarding to the COVID-19 pathogenesis, have been analyzed and discussed.

***Results:*** CoQ_10_ is an essential cofactor in the electron transport chain of the phosphorylative oxidation system. It is a powerful lipophilic antioxidant, anti-apoptotic, immunomodulatory and anti-inflammatory supplement which has been tested for the management and prevention of a variety of diseases particularly diseases with inflammatory pathogenesis. CoQ_10_ is a strong anti-inflammatory agent which can reduce tumor necrosis factor-α (TNF-α), interleukin (IL)- 6, C-reactive protein (CRP), and other inflammatory cytokines. The cardio-protective role of CoQ_10_ in improving viral myocarditis and drug induced cardiotoxicity has been determined in different studies. CoQ_10_ could also improve the interference in the RAS system caused by COVID-19 through exerting anti-Angiotensin II effects and decreasing oxidative stress. CoQ_10_ passes easily through blood–brain barrier (BBB). As a neuroprotective agent CoQ_10_ can reduce oxidative stress and modulate the immunologic reactions. These properties may help to reduce CNS inflammation and prevent BBB damage and neuronal apoptosis in COVID-19 patients.

***Conclusion:*** CoQ_10_ supplementation may prevent the COVID-19-induced morbidities with a potential protective role against the deleterious consequences of the disease, further clinical evaluations are encouraged.

## Introduction

 After the report of the first case of coronavirus disease 2019 (COVID-19) on 31 December 2019 in China,^1^ the virus rapidly spread worldwide. The World Health Organization (WHO) officially declared COVID-19 as pandemic on 11 March 2020 and millions of people have so far died from the disease.^2^ Severe acute respiratory syndrome coronavirus 2 (SARS-CoV-2), the causative agent of COVID-19, and the previously known viruses SARS-CoV and Middle East respiratory syndrome coronavirus (MERS-CoV) are known to cause threatening epidemics with severe clinical features that mostly involve the respiratory system.^3^ Coronaviridae family consists of enveloped viruses containing large positive-sense single-stranded RNA genomes which is restricted within a protein capsid and their envelope is covered with glycoprotein spikes in the shape of crowns.^2^ The spikes contain receptor binding domains and facilitate the attachment and replication of the virus.^4^ Angiotensin converting enzyme 2 (ACE2) is known as the mutual receptor for SARS-CoV-2 and its similar ancestor SARS-CoV.^5^ ACE2 is considerably expressed in the lung epithelium, and its possible role in the pathogenesis of COVID-19 has been suggested in different studies.^6^ COVID-19 is currently considered the greatest health threat internationally and there is yet no definitive treatment for the disease. Therefore, current management policy has been mainly focused on the preventive and supportive approaches.^1^ As the virus continues to rapidly spread and infect millions of people, the urge to find a definite treatment intensifies and great research attempts are being conducted to solve this global concern.

 Coenzyme Q_10_ (CoQ_10_) is a natural lipid soluble electron transporter found in the mitochondrial membrane. It is an essential cofactor in the electron transport chain of the phosphorylative oxidation system. This coenzyme molecule could undergo oxidation/reduction reactions,^7^ and act as a powerful lipophilic antioxidant, anti-apoptotic, immunomodulatory and anti-inflammatory supplement which has been tested for the management and prevention of a variety of diseases specially diseases with inflammatory pathogenesis.^8^ The role of CoQ_10_ supplementation in heart failure and neurodegenerative diseases has been well established.^8,9^ Other clinical applications of CoQ_10_ have been tested in several clinical trials on patients with inflammatory diseases such as rheumatoid arthritis, fatty liver and diabetes. CoQ_10 _supplementation has presented ameliorative effects on serum inflammatory markers.^10^ Moreover, studies on critically ill and intensive care unit (ICU) patients have revealed a severe depletion of CoQ_10_ levels. It may indicate that CoQ_10_ supplementation solely or in combination with other micronutrients like carnitine and selenium could have a considerable positive effect on disease progression and treatment outcomes.^11^ Recent studies on CoQ_10_ revealed the immunomodulatory effects of this coenzyme,^12-14^ especially in the context of viral disorders. In fact, the systemic inflammation and hypercytokinemia caused by acute viral infections may become suppressed by immunomodulatory and anti-apoptotic properties of CoQ_10_.^15-17^ Accordingly, some studies had reviewed the potential role of CoQ_10_ and other mitochondrial nutrients as therapeutic options against the systemic inflammation and mitochondrial dysfunction in COVID-19. Polymeropoulos^18^ in a brief opinion had reviewed the anti-inflammatory effects of CoQ_10_ in various clinical situation and encouraged further researches for assessing the correlation between the levels of CoQ_10_ in human body and the outcomes of COVID-19. In another comprehensive review by Pagano et al^19^ the evidences supporting the role of mitochondrial nutrients, such as α-lipoic acid, CoQ_10_ and carnitine, against acute and chronic inflammatory conditions were summarized. in this review we aimed to discuss the molecular aspects of CoQ_10_ supplementation against COVID-19. Herein, rather than the anti-inflammatory properties of CoQ_10_, we described other various pathways that CoQ_10_ supplementation could affect the disease pathogenesis including; antiviral, gene expression regulatory, immune modulatory, neuroprotective and cardiovascular protective properties of CoQ_10_. In addition, the role of CoQ10 supplementation in clinical setting particularly in critically ill patients was reviewed from a molecular point of view.

## Materials and Methods

 The current study is a narrative molecular review. Some authentic resources including PubMed, ISI, Scopus, ScienceDirect, Cochrane, and some preprint databases such as Arxiv were searched within 2000-2021. Some sensitive keywords were used to find the most relevant articles, such as molecular, CoQ_10_, viral infection, COVID-19, SARS, and corona virus infection. Finally, the selected full-text articles were reviewed and using thematic analysis, the molecular aspects of CoQ_10 _potencies against COVID-19 and other similar viral infections have been analyzed.

## Results and Discussion

###  Molecular basis of SARS-CoV-2 infection and consequent clinical characteristics of the disease

**Figure 1:**
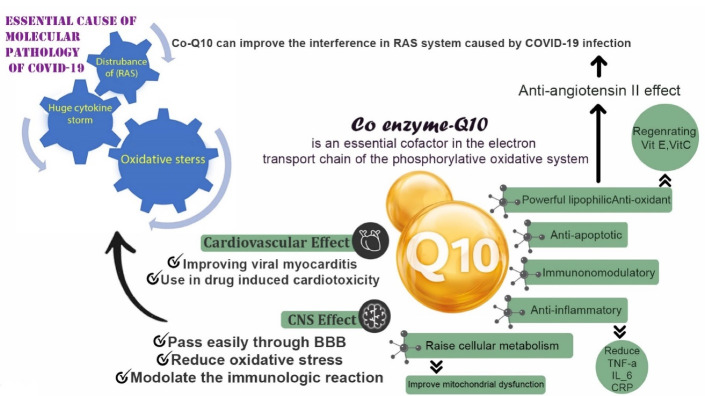
The potential protective effects of coenzyme Q_10_ against COVID-19: CoQ_10_ is an essential co-factor in electron transport chain of the phosphorylation oxidative system. Because of its lipophilic anti-oxidant power, it has potential ability for regenerating the VitE and VitC. Moreover, the anti-angiotensin 2 effect of CoQ_10_ improve the RAS activity which is upregulated in COVID-19. The anti-apoptotic, immunomodulatory, and anti-inflammatory effects of CoQ_10_ have been also confirmed through reducing TNF-a, IL-6, and CRP. The cardiovascular protective effects of CoQ_10_ have been also determined, such as improving viral myocarditis and using in drug induced cardiotoxicity. Moreover, CoQ_10_ has some protective effects in CNS. It can pass easily through BBB, reduce oxidative stress, and modulate the immunologic reaction. BBB: Brain blood barrier.

 binding of SARS-CoV-2 spike to transmembrane ACE2 is not only the first step in pathogenesis of COVID-19 but also is the most fundamental. In fact, the RNA entrance, replication and consequent cell damage is not the only pathologic concern of COVID-19.^20^ As mentioned, the viral spike protein has a strong affinity to ACE2 and this is the key point of protein interaction and further deleterious clinical and pathological properties of COVID-19.^21^ ACE2 is a transmembrane enzyme producing angiotensin_(1-7)_ and a heptamer opposing the action of angiotensin II (AngII), which is correlated with the pathogenesis of several diseases like cardiovascular, renal and fibrotic diseases. Further studies about AngII revealed that besides its vasoconstrictor effect, the immunologic, inflammatory, fibrogenic and leukocyte migratory effects are also considerable aspects of this molecular axis. AngII signaling pathway is transmitted through two G-protein-coupled receptors called AT_1_ and AT_2_. The most concerns is about ACE/AngII/AT_1_ route through which a variety of AngII adverse effects including: (1) oxidative stress production specially through NADPH oxidase enzyme upregulation, (2) inflammatory response by the activation of NF-κB translation factor and consequent tumor necrosis factor-α (TNF-α) and interleukin (IL)-6 production, (3) activating the leukocyte migratory pathway through increasing both the endothelial adhesion molecules like VCAMs and cell adhesion molecules on leukocytes, (4) contributing to endothelial dysfunction and (5) increasing the risk of arrhythmia and fibrosis through activating the proliferating pathways of fibroblasts and smooth muscle cells.^22^ Given the fact that these effects are all opposed by angiotensin_(1-7)_,^23^ the pathophysiology of SARS-CoV-2 becomes easy to understand. The viral spike protein interaction suppresses the inhibitory effect of ACE2 on the AngII system and consequently over-activates AT_1_ resulting in a propagated systemic inflammation and hypercytokinemia accompanied by immense oxidative stress in affected organs.^20^ Current studies show that the secondary hemophagocytic lymphohistiocytosis as an hyperinflammatory status, which leads to hypercytokinemia, is a common reason for death after a multiorgan failure in patients infected by COVID-19.^24^ These events are characterized by a cytokine storm due to the fulminant increased cytokines includes IL-2, IL-7, granulocyte colony stimulating factor, interferon-γ, inducible protein 10 (IP-10), monocyte chemo-attractant protein 1 (MCP1), macrophage inflammatory protein 1-α (MIP-1α), and TNF-α.^25^ In another recent work, it was demonstrated that the CD4 + and CD8 + T cells counts in the patients with severe form of COVID-19 had been reduced in negative correlation with increased IL-6, IL-10 and TNF-α, suggesting the apoptotic effect of these factors on T cells.^26^ It was also demonstrated that the apoptotic pathway triggered by IL-10, IL-6 and TNF-α passes through mitochondrial stress which is pursued by the activated caspase-9 and caspase-3.^27^ The mentioned two phenomena, systemic inflammation and oxidative stress, are the main causes of almost all clinical events in COVID-19. Pneumonia in COVID-19 is not only due to the pulmonary epithelium infection but also the increased vascular permeability, leukocyte migration and vascular hyper-inflammation play an undeniable role in the pathophysiology of the disease.^28^ The pathophysiology of cardiovascular effects of COVID-19 is not completely understood, but most researchers consider cytokine storm and myocardial inflammation as the key contributors to the events.^29^ In very recent and novel studies, the neuro-infective properties of SARS-CoV-2 have been discussed.^30-32^ Steardo et al^30^ postulated that like SARS, MERS and other members of Coronaviridae, SARS-CoV-2 could infect the CNS and PNS causing neurologic impairment. The suggested mechanism of neuro-infection in COVID-19 is hematogenous and retrograde neuronal rout invasion to CNS. Furthermore, the systemic inflammatory state could cause the neuronal damage as made in many neuro-degenerative diseases. The first study about neurological involvement in COVID-19 patients ran in Wuhan, China, reported the neurological impairment and complications including: impaired consciousness, hyposmia, hypogeusia, dizziness, headache, and cerebrovascular accidents in severely ill patients, concluding that CNS and PNS involvement are signs of poor prognosis of the disease.^31^ Li et al^33^ discussed the association of respiratory failure with neuro-invasiveness of SARS-CoV-2 and demonstrated that viral invasion to the medullary cardiorespiratory centers through the root of mechanoreceptors and chemoreceptors in lower respiratory tract could cause respiratory failure in severely ill patients. (Figure 1).

###  Biochemical and pharmacological characteristics of coenzyme Q₁₀

**Figure 2:**
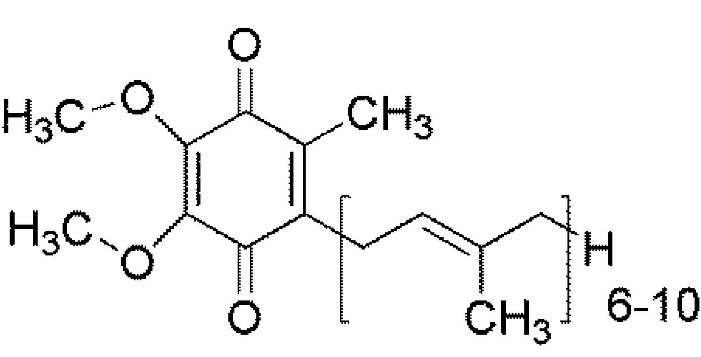
The structure of Coenzyme Q₁₀.

 In 1957 Crane et al^34^ reported the first time a quinone found in oxidized and reduced forms. Coenzyme Q₁₀ or CoQ₁₀ (2, 3dimethoxy-5methyl-6-decaprenyl benzoquinone) is a lipophilic vitamin-like compound which is also known as ubiquinone (oxidized) or ubiquinol (reduced). The chemical structure (Figure 2) consists of a benzoquinone ring connected to a long side chain containing 10 isoprene units.^35^ CoQ₁₀ is endogenously synthesized from mevalonic acid and phenylalanine.^36^ CoQ₁₀ is a well-defined physiological component of the mitochondrial respiratory chain which supports the generation of energy in the form of ATP by converting the energy in carbohydrates and fatty acids into the energy-rich adenosine triphosphate.^37^ It also reduces oxidative and nitrosative stress by decreasing the superoxide radicals and interfering with the production of peroxynitrite. CoQ₁₀ exists in two forms in the body: reduced form also known as ubiquinol is used by the body as an endogenous antioxidant, and oxidized or ubiquinone form is an electron carrier during mitochondrial respiration.^38^ Due to several features including solubility in lipid and its role in the inhibition of lipid peroxidation, CoQ₁₀ is a very effective antioxidant against the radicals produced in the biological membranes.^39^ Since the quinol form of the coenzyme Q is present in cell membranes more than the other form, it can be a very efficient antioxidant. Some enzymes such as NADH cytochrome b5 reductase, NADPH coenzyme Q reductase and NADH/NADPH oxidoreductase are effective enzymes which can keep the coenzyme Q reduced in plasma and endomembranes.^40^

 Recent studies show that CoQ₁₀ has numerous other roles including: gene expression modifying, protection of membranes and lipoproteins from protein oxidation and lipid peroxidation, and cell signaling. Therefore, these vital roles led us to its clinical application, especially in energy-demanding tissues involved by the disease, such as heart and liver.^41,42^

 CoQ₁₀ is expressed in all tissues. The body is not normally dependent on exogenous sources of CoQ_10_, but its biosynthesis is decreased with age and also many critical conditions in which the serum and tissue levels of this coenzyme are reduced by oxidative stress. In such conditions, exogenous CoQ₁₀ is required to maintain the normal blood and tissue levels.^43^ CoQ₁₀ is absorbed as a lipophilic substance and its uptake increases with high fat food. The main absorption is in the small intestine without any specific receptors. In circulation, CoQ₁₀ is reduced to ubiquinol and then taken up rapidly by the liver where CoQ₁₀ is incorporated mostly into very low-density lipoprotein (VLDL)/low density lipoprotein (LDL) particles. CoQ₁₀ supplements have poor bioavailability in oral administration due to their insolubility in water and high molecular weight.^44^ CoQ₁₀ metabolism has not been well studied in humans, but studies in animal models suggest that CoQ₁₀ is metabolized in all tissues. The main route of the elimination of CoQ₁₀ is through bile and stool excretion. A small fraction of the metabolites is phosphorylated in the cells, transported to the kidneys through blood, and excreted in the urine.^39^

###  Coenzyme Q₁₀ and medical molecular biology

 The primary physiological effect of CoQ₁₀ is described as a part of the cellular ATP synthesis system. CoQ₁₀ is a fundamental part of the oxidative phosphorylation of mitochondria. Five protein-lipid complexes situated in the inner mitochondrial membrane, which use molecular oxygen as the final electron acceptor, are engaged in oxidative phosphorylation. Complexes I–IV are responsible for the transportation of electrons to molecular oxygen. CoQ₁₀ is an electron carrier in this process. Finally, this process creates an electrochemical proton-motive force and the final complex (complex V) uses this force to form ATP.^45,46^ Further studies revealed other molecular properties of CoQ₁₀ as a powerful antioxidant, gene regulator, anti-inflammatory, and immune modulating agent which are discussed as following.

###  Coenzyme Q₁₀: a powerful antioxidant and anti-inflammatory agent

 An antioxidant is defined as a substance that inhibits or retards oxidation. CoQ_10_ is a lipid-soluble antioxidant which acts as a free radical scavenger and a membrane stabilizer, prevents phospholipid peroxidation, and regenerates vitamin E (α-tocopherol) and vitamin C (ascorbate).^47^ Kagan et al^48^ postulated that the vitamin E regenerative property of this coenzyme is a more effective pathway to reduce oxidative stress than the free radical scavenging characteristics of CoQ_10_. The preventive effect of CoQ_10_ against lipid peroxidation also plays a role as an anti-atherosclerotic property through diminishing the oxidation of LDL.^45^ CoQ_10_ also upregulates some enzymatic antioxidants like superoxide dismutase (SOD) and glutathione peroxidase.^46^ The ubiquinone (oxidized form of CoQ_10_) become reduced to ubiquinol through the enzymatic actions of NADH-cytochromeb5 reductase and NAD(P)H: quinone oxidoreductase 1.^49^

**Figure 3:**
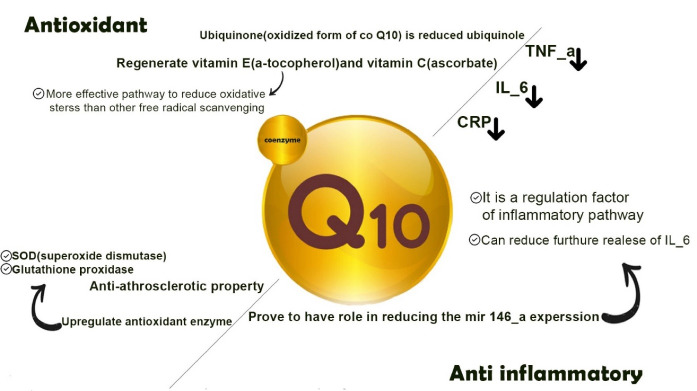
The anti-oxidant and anti-inflammatory effects of CoQ10: CoQ10 can reduce oxidative stress, through free radial scavenging methods, the best effective pathway which is performed by vitamin E (α-tocopherol); and the regeneration of this vitamin beside vitamin C (ascorbate) is also one of the CoQ10 functions. Addition to decreasing the TNF-a, IL-6, and CRP, CoQ-10 can play the role in preventing the lipid peroxidation. Meanwhile CoQ-10 can up-regulate the anti-oxidant enzymes like SOD (superoxide dismutase), and glutathione peroxidase. Moreover, it has proven that CoQ-10 can reduce the expression of mir-146, resulting in decreasing the release of IL-6 and could be a regulation factor for inflammatory pathway.

 Researches on human body revealed that the production of CoQ_10_ reduces through the ages. This contribute to the process of aging and aging related systemic inflammation.^8^ Inflammation is both the cause and the consequence of oxidative stress. CoQ_10_ as an immunomodulatory and an antioxidant could rationally act as a strong anti-inflammatory agent. Moreover, in clinic, Various meta-analysis on RCTs strongly suggest that CoQ_10 _significantly reduces TNF-α, IL-6 and C-reactive protein (CRP).^17,50^ In another study CoQ_10 _treatment proves to have a role in reducing the mir146-a expression which is a regulation factor of inflammatory pathways. Additionally, CoQ_10 _can reduce the further release of IL-6.^51^ (Figure 3)

###  Coenzyme Q₁₀: a gene expression regulator

 CoQ_10_ has been identified as a modulator of several biological processes like cell signaling and has a vital role in the mitochondrial respiratory chain and antioxidant activity.^52^ Moreover, many *in vitro *and *in vivo *studies have demonstrated that CoQ_10_, in addition to its well-known functions, affects the expression of several human genes involved in metabolism, cell signaling, nutrient transport, cell death and cell differentiation.^53^ Its diverse functions reflect its therapeutic potential as a dietary supplement for a number of diseases such as mitochondrial myopathies, migraine and cardiovascular diseases.^54^ On the other hand, the conversion of Q_10_ into its reduced form is accompanied by the generation of reactive oxygen species (ROS) which may also have an additional impact on gene expression.^52^ A study conducted by Schmelzer et al presented that the reduced form of CoQ_10_ (Q10H2) has a stronger effect on gene expression than the oxidized form CoQ_10_, primarily due to differences in bioavailability.^52^ The endogenous insufficiency of CoQ_10_ synthesis causes the up-regulation of oxidation reactions and the down-regulation of multiple genes which are crucial for growth such as RNA polymerase II. Exogenous CoQ_10_ supplies partially restore the expression of these genes; however, the expression level of another subset of genes which are involved in some biological functions such as metabolism and cell signaling is not affected by exogenous CoQ_10_ supplementation and depends solely on endogenous synthesis of CoQ_10_.^55^ It has been further demonstrated that CoQ_10_ supplementation may be effective in regulating the transcription factors contributing to inflammation and fibrosis. Pala et al^56^ had postulated that CoQ_10_ supplementation downregulate the expression of nuclear factor kappa-light-chain-enhancer of activated B cells (NF-κB) and upregulate the anti-inflammatory pathways trough enhancing inhibitors of kappa B (IκB), nuclear factor (erythroid-derived 2)-like 2 (Nrf2) and hemeoxygenase 1 (HO-1). In another study by Mohamed et al^57^ the downregulating effect of CoQ_10_ supplementation on transforming growth factor (TGF)-β1 and matrix metalloproteinase (MMP)-9 was indicated. Consistent with the above mentioned animal studies, Armanfar et al^58^ reported the downregulating effect of CoQ_10_ supplementation on IL-6, TNF-α in human subjects.

###  Coenzyme Q₁₀: an immune-modulating agent

 The effect of CoQ10 supplementation on the immune system is a complexity of direct and indirect impacts on the energy metabolism, cell signaling and gene expression of the immune cells.^59^ The role of CoQ10 in normal ATP synthesis of mitochondria could considerably prevent the cellular stress and further apoptosis in ATP depleted activated immune cells. It is notable that the majority of immune cells involving in the regulation of the immune response use the mitochondrial pathways for ATP synthesis whereas pro-inflammatory immune cells mostly use the glycolysis pathway for ATP production.^60^ In view of cell signaling and differentiation of immune cells, studies had demonstrated that CoQ10 supplementation reduced the expression of p-STAT3 and further IL-17 while upregulates the expression of p-STAT5 and further FOXP3 which leads to differentiation of CD4 + T cells into regulatory T cells rather than the pro-inflammatory T helper 17 cells.^61^ In one in-vitro study, the peripheral blood mononuclear cells were exposed to CoQ₁₀ for 24 h and the secretion of some cytokines was examined including IL-1β, IL-1RA, IL-6, IL-10, IL-2, INF-γ, TNF-α and IL-2. As a result, only TNF-α and IL-2 secretion was significantly decreased. Notably the outcome of this experiment presented that the treatment with an average concentration of 1.25µM CoQ₁₀ had the best effect, and the higher levels (up to 10µM) did not exert a significant difference.^53^ The different behavior of CoQ₁₀ in lower and higher dosages is suggestive of a biphasic role for CoQ₁₀. Moreover, in another study by Gollapudi and Gupta, CoQ₁₀ presented an apoptotic protective effect on CD4 + and CD8 + which had been induced with an oxidative stressor. The selected T cells after treatment with CoQ₁₀ (under 10µM concentration) for 24 h showed a strong resistance to an oxidative stress-induced mitochondrial apoptotic pathway. It represented that CoQ₁₀ could inhibit the activation of both caspase-9 and caspase-3 in apoptotic cascades. Moreover, it can reduce the production of ROS and prevent the oxidative stress-induced mitochondrial membrane depolarization in CD4 + and CD8 + T cells.^14^ Another critical step in apoptotic process that is also suppressed by CoQ₁₀ is cytochrome C release from mitochondria according to some experimental studies.^62^ Furthermore, some studies demonstrated a lowered TNF-α secretion and a significant declined secretion of MIP-1α, MCP1 with CoQ₁₀ treatment on THP-1 cell line, the events which are very important in the COVID-19-induced hyper-inflammatory state. Since the monocytes are able to convert oxidized CoQ₁₀ into its reduced form, this reduction can be justified. NF-κB is a transcription factor for many genes involved in immune responses including which encode MIP-1α, MCP1, and TNF-α. It is believed that down-modulation of these factors is due to NF-κB inhibition. It has not been clearly confirmed that how CoQ₁₀ effects on NF-κB, but there are some evidences for NF-κB inhibition by antioxidant compounds^63,64^ (Figure 3).

###  Coenzyme Q₁₀: an antiviral nutrient

 The viral infections, caused by RNA or DNA virus, trigger the production of reactive species (RS) and ROS including: NO, O2^.^-, OH^•^ and their by-products (such as H2O2), which interfere with normal functions of the infected cells such as gene expression and metabolism. As an instance, a higher RS level in the host cell promotes the activating of NF-κB which can lead to increased viral replication.^65^ There are also some evidences that antioxidant agents can mediate viral pathogenesis through the reinforcement of cell resistance against oxidative stress. Moreover, it has been determined that the antioxidant agents exert an important role in decreasing the replication of RNA viruses such as flaviviruses, alphaviruses, and Japanese encephalitis virus through the various pathways in different stages.^66^ Moreover, a recent molecular docking study of various quinone derivatives demonstrated that CoQ10 may indicate direct antiviral properties as CoQ10 could effectively binds to the viral protein PDB 6Y84 protease of SARS-COV-2.^67^

###  Coenzyme Q₁₀ and anti-angiotensin II properties

 The renin–angiotensin system (RAS) has been shown to play a vital role in physiological and pathophysiological events in cardiovascular system. In this cascade, ACE converts AngI to AngII, and AngII as the prime component of RAS, disrupts endothelial function by increasing the oxidative stress. On the other hand, AngII and its receptor induce the activation of NADPH oxidase whereby the synthesis of ROS is increased. When the local levels of ROS are increased, a considerable cellular damage and oxidative stress will occur by interaction with cell membranes, DNA and other molecules.^68^

 Some experimental studies presented that CoQ₁₀ is involved in enhancing the expression of the antioxidant enzymes and eliminating the free radicals. Treatment with antioxidant agents may remove the misbalance of RAS caused by oxidative stress.^69^ Moreover, studies demonstrated the preventive effect of CoQ_10_ against angiotensin induced up-regulation of NADPH oxidase enzyme.^70^

###  Clinical implications of coenzyme Q₁₀ in COVID-19

 CoQ₁₀ has been the subject of interest in a variety of diseases including cardiovascular, neurodegenerative, kidney and systemic inflammatory diseases.^8^ The antioxidant, anti-inflammatory, immunomodulatory and gene expression regulator properties of this molecule highlight its application as a considerable choice for nutrient therapy in the aforementioned diseases.^47^ Some of these conditions such as cardiovascular diseases, hyper-inflammatory state and critical stages of illnesses like septic shock share some features with COVID-19 in pathophysiology. The following statements describe this shared features and possible effects of CoQ_10_ supplementation in COVID-19 patients.

###  Coenzyme Q₁₀ and its potential cardioprotective effects

 CoQ₁₀ has a pivotal role in myocyte bioenergetics, exerts anti-inflammatory effects, and reduces oxidative stress. CoQ_10_ supplementation could be beneficial for a wide spectrum of cardiovascular diseases including: heart failure, hypertension, myocardial infarction, viral myocarditis, arrhythmias, and drug-induced or idiopathic cardiomyopathies.^71^

 The action mechanism of this supplement, according to Greenberg and Ferishman,^69^ is to not only enhance the cellular aerobic metabolism but also exert cardiovascular effects including: the modification of endothelial dysfunction, preserving the function of the NA ^+^ /K ^+^ ATPase, stabilizing the cellular membrane, reducing blood viscosity, modulating the immune system, and suppressing systemic inflammation. CoQ_10_ could be helpful for cardiac patients as a supplement that contribute to increasing mitochondrial phosphate/oxygen ratio, alleviating reperfusion injury after hypoxic conditions, modifying QRS duration abnormalities, and improve NYHA function class.^72^ Moreover, CoQ_10_ improves extracellular SOD and flow-mediated-dilation,^72^ and protect against progressive left ventricular remodeling and fibrosis.^73^ Ultimately, CoQ_10 _can reduce total cardiac events and could be protective against myocardial infraction, congestive heart failure, and dilated and drug-induced types of cardiomyopathies.^74^

 The protective role of CoQ_10_ in improving viral myocarditis and drug induced cardiotoxicity introduces this supplement as an appropriate choice for the prevention of COVID-19 cardiovascular complications which is generally influenced by two factors: cytokine storm, and adverse effects of the medications.^29^ The hypercytokinemia caused by SARS-COV-2 infection could lead to fulminant myocarditis,^75^ a lethal condition mostly caused by hyper-inflammatory state and cytokine storms, particularly during a viral infection.^76^ Evidence has demonstrated that the blood levels of inflammatory cytokines in critically ill patients in the ICU are higher than the patients not admitted to the ICU; additionally, the level of IL-6 has shown to be higher in patients with cardiac injury.^75^ The anti-inflammatory, antioxidant, and immunomodulatory effects of CoQ_10_ could suppress the hyper-inflammatory state, particularly through reducing IL-6, TNF-α and other inflammatory cytokines resulting in the prevention of cardiovascular events in COVID-19 patients and the alleviation of the cardiac complications caused by the cytokine storm in this disease.^74,77^

 Despite the fact that no definitive treatment for COVID-19 has yet been discovered, several curative and supportive medications have been suggested; the most fundamental of which include chloroquine, hydroxyl chloroquine, remdesivir, and potent antibiotics preventing bacterial super infections.^78,79^ Among the adverse effects of these drugs, particularly hydroxyl chloroquine, cardiovascular complications are of great importance.^80^ These drugs induce cardiotoxicity through increasing oxidative stress, triggering endothelial dysfunction and elevating tissue inflammation.^81^ CoQ_10_ counteracts the cardio-toxic effects of these drugs by improving the mechanism of oxidative phosphorylation, reducing oxidative stress and decreasing the inflammation of the myocardium.^82^

###  Coenzyme Q₁₀, primary hypertension and endothelial dysfunction

 The pathophysiology of primary hypertension is generally associated with the oxidative stress in the endothelium which leads to decreased available NO for the cells of vascular intima layer, mitochondrial dysfunction of the endothelium, and eventually endothelial dysfunction.^83^ CoQ_10_ could improve hypertension through decreasing vascular oxidative stress, improving the function of mitochondria, moderating the effects of AngII, and reducing the level of Aldosterone.^71^ As mentioned, in the pathophysiology of COVID-19, the RAS system is disturbed due to the interference caused by the protein-protein interaction of the virus spikes and ACE2, leading to the down-regulation of ACE2 which could enhance the pathologic effects of AngII and frustrate the AngII/Ang_(1-7)_ ratio resulting in the severe complications of COVID-19 disease.^21^ Supplementation with Q10 could improve the interference in the RAS system caused by COVID-19 infection through exerting anti-AngII effects and decreasing oxidative stress.^70^ The antihypertensive effects of CoQ_10_ are not entirely confirmed and more well-designed clinical trials are suggested to confirm it.^84^ Studies have demonstrated that CoQ_10_ could not solely reduce blood pressure but could be beneficial against hypertension in the context of metabolic diseases like diabetes as an adjunctive therapy to adjust blood pressure.^82^

###  Coenzyme Q₁₀ in critically ill and ICU patients

 The molecular and cellular mechanism of sepsis has not been entirely discovered and includes different aspects. One of the most crucial elements of sepsis is severe oxidative stress accompanied by the mitochondria dysfunction.^85^

 The decreased levels of CoQ_10_ during septic shock have also a significant importance.^86^ The elevated levels of IL-6 and IL-8 which have a negative relationship with CoQ_10_ levels, and the decreased LDL that is the plasma carrier of the coenzyme lead to lower CoQ_10_ levels during the occurrence of septic shock.^87^ The coenzyme inversely correlates with vascular endothelial biomarkers like VCAM and inflammatory cytokines like IL-10, and its decrease during septic shock, contributes to the organ failure related to the mitochondrial dysfunction.^86^

**Figure 4:**
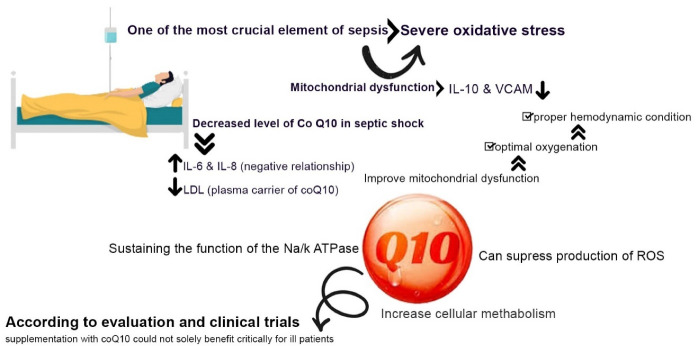
The effective role of CoQ_10_ against severe oxidative stress by improving the mitochondrial dysfunction: One of the most crucial elements of sepsis infections is severe oxidative stress which is caused by mitochondrial dysfunction leading to decreasing IL-10 and VCAM. During a septic shock, a condition in which the level of CoQ_10_ is decreased and it accompanies by increasing IL-6 and decreasing LDL (plasma carrier of CoQ_10_), optimal oxygenation and as a result making proper hemodynamic condition, are supposed to improving the mitochondrial dysfunction. Although CoQ_10_ has proven benefits in suppressing the production of ROS, raising the cellular metabolism, and sustaining the function of Na/K ATPas, the supplementation with CoQ_10_ could not solely benefit critically for the ill advanced patients. ROS: Reactive oxygen species

 Meanwhile, despite the providing stable and proper hemodynamic conditions along with the optimal oxygenation in the critically ill patients, death rates could not be reduced in these people. This clearly indicates that the substantial mitochondrial dysfunction in the critically ill patients prohibits using the oxygen to produce intracellular ATP even with proper oxygenation.^88^ CoQ_10_ could not only counteract the oxidative stress in sepsis as a strong mitochondrial and membranous antioxidant, but could also suppress the production of ROS, increase cellular metabolism and enhance the patient’s response to oxygenation by alleviating the mitochondrial dysfunction through stabilizing the plasma membrane, sustaining the function of the NA ^+^ /K ^+^ ATPase, and regulating the oxidative phosphorylation system. Nevertheless, according to evaluations and clinical trials, supplementation with CoQ_10_ could not solely benefit critically ill patients and it is advised to prescribe CoQ_10_ with Selenium as a crucial component of several metabolic enzymes and selenoproteins.^11^ (Figure 4).

###  Coenzyme Q10 and its potential neuroprotective effects

 Most of neurodegenerative diseases like Alzheimer and Parkinson disease, despite their exclusive neurologic and molecular properties, share some common pathological aspects such as neuro-inflammation, excitotoxicity cascade induced neuronal apoptosis, and mitochondrial dysfunction in affected neurons.^89^ CoQ_10_ is a nutrients of interest in adjunctive therapy and the prevention of these types of age related diseases.^90^ CoQ_10_ with anti-inflammatory, antioxidant and immunomodulatory properties, could suppress the CNS inflammation in such diseases in addition to reducing oxidative stress and enhancing mitochondrial function.^91^ CoQ_10_ could also prevent neuronal apoptosis trough keeping mitochondrial permeability transition pores in closed conformation and blocking the apoptosis pathway induced by N-methyl D-aspartate (NMDA) glutamate receptors or non-NMDA glutamate receptors.^92^

 The suggested pathophysiology of neurologic involvement in COVID-19 patient is based on three events; a retrograde trans-synaptic infection of CNS, hematogenous infection of CNS in the context of disrupted BBB due to hypercytokinemia and a systemic inflammation which causes both endothelium and astrocytes dysfunction in BBB, and the direct impact of systemic inflammation and oxidative stress on CNS and PNS causing neuronal damage and pathologic reactions in the supportive tissue of neurologic system, blood vessels, coagulation cascades and endothelium resulting in the cerebrovascular accidents.^30,32^

 CoQ_10_ as a lipophilic antioxidant which passes easily through BBB, has a direct effect on reducing oxidative stress and modulating the immunologic reactions, which could be beneficial through suppressing the systemic inflammation,^90^ preventing BBB damage,^93^ and neuronal apoptosis in COVID-19 patients.^92^ Accordingly, CoQ_10_ supplementation could prevent the developing CNS and PNS damage and further deleterious consequences like central respiratory failure, delirium and loss of conciseness, leading to a permanent brain injury and death^31^ (Figure 1).

## Conclusion

 COVID-19 as a pandemic lethal infection, currently, has no definite treatment. The interaction of virus-spike with ACE2 receptor leads to the down-regulation of ACE2 which could enhance the pathologic effects of AngII and disturb the AngII/Ang_(1-7)_ ratio. It could result in a huge cytokine storm, and an extensive oxidative stress which are the molecular basis of the most complications induced by COVID-19. CoQ_10_ as an essential electron transporter in the phosphorylative oxidation system is a powerful lipophilic antioxidant, anti-apoptotic, immunomodulatory and anti-inflammatory supplement which has been tested for the management and prevention of a variety of diseases specially diseases with inflammatory pathogenesis. CoQ_10_ can decrease the important inflammatory cytokines and prevent the organ damages due to a huge oxidative stress. CoQ_10_ can be also a cardio-protective and neuroprotective agent through reducing the viral toxicity against cardiomyocytes and CNC neurons. Accordingly, CoQ_10_ supplementation could prevent the COVID-19-induced morbidities and has a potential protective role against the deleterious consequences of the disease.

## Competing Interests

 All authors declare no conflict of interest.

## Ethical Approval

 Not applicable.
